# COMPARATIVE EVALUATION OF MAGNETIC RESONANCE CHOLANGIOPANCREATOGRAPHY AND PERIOPERATIVE CHOLANGIOGRAPHY IN PATIENTS WITH SUSPECT CHOLEDOCHOLITHIASIS

**DOI:** 10.1590/0102-672020180001e1416

**Published:** 2019-01-07

**Authors:** Simone Reges PERALES, Luiz Ronan Marquez Ferreira SOUZA, Eduardo CREMA

**Affiliations:** 1 Discipline of Digestive System Surgery;; 2 Discipline of Radiology and Diagnostic Imaging;; 3 Program of Post-Graduation in Health Sciences, Federal University of the Triângulo Mineiro), Uberaba, MG, Brazil

**Keywords:** Cholangiopancreatography, magnetic resonance, Cholangiography, Choledocholithiasis, Gallstones, Cholelithiasis., Colangiopancreatografia por ressonância magnética, Colangiografia, Coledocolitíase, Cálculos biliares, Colelitíase.

## Abstract

**Background:**

Cholelithiasis is a highly prevalent condition, and choledocholitiasis is a high morbidity complication and requires accurate methods for its diagnosis.

**Aim:**

To evaluate the population of patients with suspected choledocholitiasis and check the statistical value of magnetic resonance cholangiopancreatography, ultrasonography, the laboratory and the clinic of these patients comparing them to the results obtained by perioperative cholangiography.

**Methods:**

This is a retrospective cohort study, which were evaluated 76 patients with cholelithiasis and suspected choledocholithiasis.

**Results:**

It was observed that the presence of dilatation of the biliary tract or choledocholithiasis in the ultrasonography was four and eight times increased risk of perioperative cholangiography for positive choledocholithiasis, respectively. For each unit increased in serum alkaline phosphatase was 0.3% increased the risk of perioperative cholangiography for positive choledocholithiasis. In the presence of dilatation of the bile ducts in the ultrasonography was four times greater risk of positive magnetic resonance cholangiopancreatography for choledocholithiasis. In the presence of pancreatitis these patients had five times higher risk of positive magnetic resonance cholangiopancreatography for choledocholithiasis. On the positive magnetic resonance cholangiopancreatography presence to choledocholithiasis was 104 times greater of positive perioperative cholangiography for choledocholithiasis.

**Conclusions:**

The magnetic resonance cholangiopancreatography is a method with good accuracy for propedeutic follow-up for the diagnosis of choledocholithiasis, consistent with the results obtained from the perioperative cholangiography; however, it is less invasive, with less risk to the patient and promote decreased surgical time when compared with perioperative cholangiography.

## RESUMO

**Racional:**

A colelitíase é afecção de alta prevalência, sendo a coledocolitíase complicação de elevada morbidade e que necessita de métodos acurados para seu diagnóstico.

**Objetivo:**

Avaliar o perfil populacional de pacientes com suspeita de coledocolitíase e verificar o valor estatístico da colangiopancreatografia por ressonância magnética, da ultrassonografia, dos exames laboratoriais e da clínica desses pacientes e compará-los aos resultados obtidos pela colangiografia peroperatória.

**Métodos:**

Trata-se de estudo longitudinal, de coorte, retrospectivo, no qual foram avaliados 76 pacientes com diagnóstico de colelitíase e suspeita de coledocolitíase.

**Resultados:**

Observou-se que na presença de dilatação das vias biliares ou coledocolitíase na ultrassonografia havia risco quatro e oito vezes maior, respectivamente, de colangiografia peroperatória positiva para coledocolitíase. Para cada unidade de aumento na fosfatase alcalina sérica houve aumento em 0,3% no risco de colangiografia peroperatória positiva para coledocolitíase. Na presença de dilatação das vias biliares na ultrassonografia ou clínica de pancreatite havia risco quatro e cinco vezes maior, respectivamente, de colangiopancreatografia por ressonância magnética positiva para coledocolitíase. Na presença de colangiopancreatografia por ressonância magnética positiva para coledocolitíase o risco foi 104 vezes maior de colangiografia peroperatória positiva para coledocolitíase.

**Conclusão:**

A colangiopancreatografia por ressonância magnética para seguimento propedêutico tem boa acurácia para o diagnóstico de coledocolitíase, e concordante com os resultados obtidos na colangiografia peroperatória. O método é menos invasivo, com menores riscos ao paciente e com diminuição do tempo cirúrgico dispendido para realização da colangiografia peroperatória.

## INTRODUCTION

Cholelithiasis is a condition of high prevalence, mainly in the Western population detected in 6% of men and 9% of American women^5^. In Brazil, studies on its prevalence are scarce, but by means of ultrasound examination (USG) it is 9.3% in individuals over 20 years of age and, therefore, it is estimated that around 10 million Brazilians from the second decade of life have cholelithiasis^2^. In most cases, cholelithiasis is asymptomatic and will remain so throughout life, with approximately 20% of these patients developing symptoms after 15 years^8^ ^,^ ^20^.

Choledocholithiasis is defined as the presence of gallstones in the bile ducts, with an estimated prevalence of 5-20% of patients undergoing cholecystectomy, with a higher incidence according to age^15^. Based on the high incidence and complications of high morbimortality of choledocholithiasis, methods that confirm or exclude the presence of gallstones in the bile duct in order to establish the best propaedeutic approach become paramount. They will be both better and less invasive to the patient, more accurate and less costly^21^.

The objective of this study was to analyze patients undergoing cholecystectomy with suspected choledocholithiasis and to compare the efficacy of magnetic resonance cholangiopancreatography (MRCP) with perioperative cholangiography (CPO). Also, to evaluate the presence of choledocholithiasis or dilatation of the biliary tract in the preoperative USG; preoperative laboratory abnormalities; and clinical parameters as predictors of choledocholithiasis in the evaluation compared to CPO.

## METHODS

The present study was submitted and approved by the Ethics and Research Committee of the Federal University of the Triângulo Mineiro. This was a longitudinal retrospective cohort study in which patients with cholelithiasis with signs or symptoms that suggested the possibility of choledocholithiasis in the Department of Digestive System Surgery of the Hospital de Clínicas of the Federal University of Triângulo Mineiro, Uberada, Brazil, in the period between August 16, 2012 and May 22, 2015.

Inclusion criteria were based on the selective use of CPO^19^: cholecystitis, pancreatitis or jaundice up to three months before cholecystectomy; alkaline phosphatase (AF), gamma-glutamyltransferase (GGT), total bilirubin (BI), amylase (AMIL), aspartate aminotransferase (AST) or alanine aminotransferase (ALT) laboratory tests up to two weeks prior to cholecystectomy; USG of abdomen with dilatation of bile ducts and/or visualization of common bile duct calculus up to two weeks before cholecystectomy; cholelithiasis in patients over 65 years of age.

The exclusion criteria were: not agreeing to participate in the survey; conditions that offered technical difficulty to perform MRCP (claustrophobia, weight above 130 kg and patients with pacemakers); inconsistent data in the medical record.

 Selected patients underwent preoperative MRCP and subsequently underwent cholecystectomy with CPO. The examinations were performed with the Siemens Acanto^®^ device of 1.5 Tesla, with Body-Flex coil. All the exams had the reports issued by a single radiologist specializing in abdominal resonance.

CPOs were performed in the operating room during cholecystectomy and were based on the technique described by Mirizzi^5^, preceded by administration of muscle relaxant (hyoscine/glucagon) and using warm iodinated contrast (30% concentration), injected through a clear catheter positioned in the cystic duct.

The following variables were evaluated: age; gender; result of MRCP; result of the CPO; biliary tract dilatation in the USG; evidence of choledocholithiasis in the USG; history of jaundice, cholecystitis or pancreatitis; laboratory exams.

### Statistical analysis

A descriptive analysis was carried out through the preparation of frequency tables of categorical variables, with absolute (n) and relative (%) frequency values ​​and descriptive statistics of the numerical variables with presentation of the mean, standard deviation, minimum, maximum and median values. Kappa coefficient was applied as a measure of agreement. Mann-Whitney test was used to compare numerical variables between the groups. The chi-square test was used to compare proportions. To identify factors that discriminate the positive CPO, the univariate and multiple logistic regression analysis with Stepwise criterion of variable selection was used. The level of significance adopted for this study was 5%. Statistical analysis was performed using SAS System for Windows (Statistical Analysis System), version 9.4. SAS Institute Inc, 2002-2012, Cary, NC, USA

## RESULTS

A total of 76 patients were included, of which 27 (36%) were men and 48 (6%) women. The mean age was 47.7±19.1 years. Sixty-three underwent USG of the abdomen, and in 14 (22.2%) the presence of choledocholithiasis was evidenced and in 18 patients (28.6%) there was dilatation of the biliary tree.

Seventy-two patients underwent MRCP, being positive for choledocholithiasis in 27 (37.5%) and negative in 45 (62.5%); and 76 patients performed CPO, being positive in 20 (26.3%) and negative in 56 (73.7%).

The relationship between CPO and categorical variables MRCP, biliary tract dilatation to the USG, choledocholithiasis to the USG, cholecystitis and pancreatitis were analyzed by chi-square and Kappa test in order to evaluate agreement; the data are summarized in Table 1. There was good concordance between the results of MRCP and choledocholithiasis to the USG in relation to CPO. The MRCP presented positive predictive value (PPV) of 70.37%, negative predictive value (NPV) of 97.78%, sensitivity of 95% and specificity of 84.62%, with an accuracy of 87.5%.

**TABLE 1:** Association between categorical variables and perioperative cholangiography

Exam	Positive predictive value	Negative predictive value	Sensitivity	Specificity	Accuracy	Kappa
MRCP	70.37%	97.78%	95.00%	84.62%	87.50%	Good agreement
Dilatation of biliary tract to USG	50%	80%	50%	80%	71.40%	No agreement
Choledocholithiasis to the USG	64.29%	81.63%	50%	88.89%	77.80%	Good agreement
Cholecystitis	16.67%	71.88%	10%	82.14%	63.2%	No agreement
Pancreatitis	9.52%	67.27%	10%	66.07%	51.3%	No agreement

According to the univariate logistic regression analysis of the MRCP factors, biliary tract dilatation and choledocholithiasis to the USG, cholecystitis, pancreatitis and laboratory tests associated with the risk of choledocholithiasis in the CPO, was observed: a) for MRCP, OR=104,488 and <0.0001, indicating that in the presence of MRCP positive for choledocholithiasis there was a 104-fold higher risk of choledocholosis positive for choledocholithiasis in the same patient; b) in relation to the dilatation of the bile ducts to the USG, OR=4,000 and p=0.002, indicating that in the presence of dilatation of the bile ducts to the USG there was a four-fold increased risk of positive choledocholithiasis; c) choledocholithiasis to USG, OR=8,000 and p=0.0019 indicating that in the presence of choledocholithiasis there was an eight-fold increased risk of choledocholithiasis in CPO; d) in relation to AF, was obtained OR=1.003 and p=0.0321 indicating that for each unit of its increase the risk of positive choledocholithiasis was 0.3%.

Studying the relationship between MRCP and the categorical variables CPO, dilatation of biliary tract to the USG, choledocholithiasis to the USG, cholecystitis and pancreatitis through the chi-square and Kappa test in order to evaluate agreement, was observed the data summarized in Table 2. There was good agreement between the results of CPO and dilation of biliary tract to the USG in relation to MRCP. CPO had 95% PPV, NPV 84.62%, sensitivity of 70.37% and specificity of 97.78%, with an accuracy of 87.5%.

**TABLE 2:** Association between categorical variables and MRCP

Exam	Positive predictive value	Negative predictive value	Sensitivity	Specificity	Accuracy	Kappa
CPO	95.00%	84.62%	70.37%	97.78%	87.50%	Good agreement
Bile duct dilation USG	76.47%	72.73%	52.00%	88.89%	73.80%	Good agreement
Choledocholithiasis USG	71.43%	68.09%	40.00%	88.89%	68.8%	No agreement
Cholecystitis	16.67%	58.33%	7.41%	77.78%	51.40%	No agreement
Pancreatitis	14.29%	52.94%	11.11%	60.00%	41.7%	No agreement

According to a univariate logistic regression analysis of the CPO factors, biliary tract dilatation and choledocholithiasis to the USG, cholecystitis, pancreatitis and laboratory tests (AF, GGT, BI, Amil, AST and ALT) associated with the risk of choledocholithiasis in MRCP were: a) regarding CPO, OR=104.488 and p<0.0001 were obtained, indicating that in the presence of positive CPO for choledocholithiasis there was a 104-fold higher risk of MRCP positive for choledocholithiasis in the same patient; b) in relation to biliary tract dilatation at the USG, OR=8.667 and p=0.0012 were obtained, indicating that in the presence of biliary tract dilatation at the USG there was an eight-fold increased risk of MRCP positive for choledocholithiasis; c) regarding choledocholithiasis at USG, OR=5.332 and p=0.0124, were obtained indicating that in the presence of choledocholithiasis at USG there was a five-fold increased risk of choledocholithiasis positive MRCP; d) in relation to pancreatitis, OR=5.332 and p=0.0144 were obtained, indicating that in the presence of pancreatitis there was a five-fold increased risk of MRCP positive for choledocholithiasis.

The variables cholecystitis, AF, GGT, BI, Amil, AST and ALT showed no statistical value in the univariate analysis.

## DISCUSSION

Cholelithiasis affects more than 20 million Americans in adulthood at an annual cost of $ 6.2 billion^6^. Approximately 15% of patients may develop complications with high potential for morbidity and choledocholithiasis, which often requires the use of invasive methods for their definitive diagnosis^7^. Recurrent symptoms, acute pancreatitis or acute cholangitis are common^19^.

In patients with suspected choledocholithiasis based on history and physical examination, laboratory tests are initially requested and imaging is initiated through abdominal USG. Subsequently, other imaging modalities can be used, such as MRCP, CPO and endoscopic retrograde cholangiopancreatography (ERCP), depending on the availability of these exams and factors related to the patient^12^.

In this study, 76 patients with a diagnosis of cholelithiasis and suspected choledocholithiasis with an epidemiological profile consistent with the literature were analyzed. One of the objectives of the present study was to evaluate the relationship of the initial clinical presentation of the patients with the results obtained in the CPO and MRCP, in order to evaluate whether the observed clinical manifestation could be used as an indicator of the presence of choledocholithiasis. After statistical evaluation with concordance tests and comparison of proportions, the presence of cholecystitis and acute pancreatitis did not show concordance with the results obtained in the CPO or MRCP, such that they should not be used as predictors of a positive outcome for choledocholithiasis. On the other hand, in a univariate analysis comparing the pancreatitis clinic with the MRCP result, a five-fold increased risk of MRCP positive for choledocholithiasis was observed in the presence of pancreatitis.

In the literature, laboratory tests of the liver profile have a high negative predictive value, reported in up to 97%, with a positive predictive value of 15%^23^. In the present paper, studying the relationship between the variables AF, GGT, BI, Amil, AST and ALT with the result obtained in the CPO and the MRCP, no association was observed between them, concluding that the changes in these tests could not predict the appearance calculi in the CPO and MRCP. However, in the logistic regression analysis, AF compared to CPO showed an association with the risk of choledocholosis positive for choledocholithiasis, being an independent predictor of choledocholithiasis. The literature shows similar data, and for AF elevations the sensitivity for choledocholithiasis reported is 57% and the specificity is 86%, with OR=2,010.

The USG sensitivity for choledocholithiasis ranges from 20-90%^14^, with a specificity of 91%^11^. Comparing the presence of choledocholithiasis to the USG of the abdomen and the positive result for choledocholithiasis in the CPO, a good concordance was observed between the results and eight times the risk of choledocholithiasis. Regarding the choledocholithiasis present to the USG of the abdomen compared to the positive result in MRCP, there was no concordance between the results, but a five-fold increase in MRCP positive for choledocholithiasis was observed in the same patient.

Thus, there was good agreement between the choledocholithiasis identified at the USG and the positive result at the CPO and, also, an increase in the risk of positive MRCP was observed when choledocholithiasis was visualized at the USG.

Bile duct dilation is considered in the literature as a diameter greater than 6 mm to the USG is suggestive, but not specific, of choledocholithiasis. USG of the abdomen has a sensitivity of 77-87% for detection of biliary tract dilatation, frequently associated with choledocholithiasis^1^. Comparing the biliary dilation visualized to the USG to the CPO result, specificity of 80% was found; sensitivity of 50%; accuracy of 71.4% and Kappa of 0.30. Therefore, in the current study, there was no agreement between the results. On the other hand, in the univariate analysis, the dilatation of the biliary tract to the USG compared to the CPO positive for choledocholithiasis presented OR=4,000, with a value of p=0.002, indicating that in the presence of dilatation of the biliary tract to the USG there was a fourfold risk of CPO positive for choledocholithiasis. Regarding the dilation of bile ducts to the USG compared to the results of the MRCP, the following results were found: specificity 88.89%; sensitivity 40%; accuracy 68.8%; and Kappa of 0.3087, indicating good agreement among the methods. In univariate analysis, OR=8.667 was evaluated, with p=0.0012 indicating that in the presence of biliary tract dilatation at the USG there was an eight-fold increased risk of MRCP positive for choledocholithiasis. Therefore, there was good agreement between the biliary tract dilatation identified to the USG and the positive result in the MRCP and, also, an increase in the risk of positive CPO was observed when visualizing dilatation of the biliary tract to the USG.

Based on the initial laboratory and ultrasonographic evaluation, patients with cholelithiasis may be, among other forms that attempt to estimate the risk of choledocholithiasis, stratified as low risk, intermediate risk and high risk of choledocholithiasis^14^. For low-risk patients, cholecystectomy without additional diagnostic exams is accepted. For those at high risk, prior to cholecystectomy, it is recommended to perform ERCP for bleaching of the bile duct. It has the advantage of being a diagnostic and therapeutic method, with sensitivity for choledocholithiasis estimated between 80-93%, specificity of 99-100%, but not yet widely available in Brazil^14^. Still, it is an invasive examination, which requires specific technical knowledge, and is associated with complications such as pancreatitis and perforations^9^.

The debate arises as to how to proceed with the evaluation of patients in the intermediate risk group, in which preoperative assessment through MRCP or endoscopic or perioperative USG can be indicated through CPO^14^. However, due to the risk associated with the manipulation of the biliary tract with ERCP or CPO, it is necessary to validate a non-invasive method for the diagnosis of choledocholithiasis.

Thus, the present study aimed to compare the efficacy of choledocholithiasis among the two main methods used in the intermediate risk group for choledocholithiasis: MRCP and CPO.

Here, 72 patients underwent MRCP, being positive for choledocholithiasis in 27 patients (37.5%) and negative in 45 (62.5%); and 76 patients performed CPO, being positive in 20 (26.3%) and negative in 56 patients (73.7%).

In the literature, CPO can be successfully completed in 88-100% of cases, with reported sensitivity of 59-100% and specificity of 93-100% for choledocholithiasis. Generally, it requires between 10-17 min to be performed in laparoscopic cholecystectomy^13^. However, it is highly operator dependent and is not routinely performed by many surgeons^10^. However, it is a safe diagnostic method when performed selectively^18^.

MRCP, a non-invasive method of bile duct imaging, has a sensitivity described between 85-92% and specificity of 93-97% for choledocholithiasis^17^. When used to exclude the presence of choledocholithiasis, it may exclude CPO in order to avoid possible injuries to the bile ducts resulting from the use of this invasive method, in addition to shortening the operative time^4^.

In the present study, comparing the positivity of MRCP to choledocholithiasis with the result also positive for choledocholithiasis obtained in the CPO, the following results were obtained: specificity 84.62%; sensitivity 95%; 87.5% accuracy and Kappa 0.7188. Therefore, there was good agreement of the results obtained in the preoperative period through MRCP and those obtained in the postoperative period through CPO. In the logistic regression analysis, OR=104.488 with p<0.0001, indicating that the presence of MRCP positive for choledocholithiasis was associated with a 104-fold higher risk of choledocholine-positive CPO in the same patient.

Therefore, through the data obtained, it can be concluded that it is safe to indicate both CPO and MRCP in cases suspected of choledocholithiasis through the initial clinical, laboratory and ultrasound, since the two methods are concordant in their results. CPO is invasive technique, however, with a lower incidence of pancreatitis when compared to ERCP, reported in 1% and 5.4%, respectively^16^. A false-positive rate of CPO is reported in the literature between 2-16%, due to air bubbles that mimic calculus or contrast failure in progressing to the duodenum, which may result in unnecessary interventions of the biliary tree as well as iatrogenic lesions coming from this manipulation^22^. In addition, it is a method that prolongs operative time, exposes the patient to ionizing radiation and to complications related to contrast.

## CONCLUSION

Comparing preoperative MRCP and CPO, there was good agreement between the two exams. The two methods are concordant in their results, in the case of patients with intermediate risk of choledocholithiasis, in whom there is no formal indication for ERCP, and thus, it is preferable to indicate MRCP as a method of diagnostic choice because it is a less invasive and less potential for complications.
